# Heterojunction Engineering Enhanced Self‐Polarization of PVDF/CsPbBr_3_/Ti_3_C_2_T*_x_* Composite Fiber for Ultra‐High Voltage Piezoelectric Nanogenerator

**DOI:** 10.1002/advs.202300650

**Published:** 2023-05-11

**Authors:** You Xue, Tao Yang, Yapeng Zheng, Kang Wang, Enhui Wang, Hongyang Wang, Laipan Zhu, Zhentao Du, Hailong Wang, Kuo‐Chih Chou, Xinmei Hou

**Affiliations:** ^1^ Institute for Carbon Neutrality University of Science and Technology Beijing 100083 Beijing China; ^2^ State Key Laboratory of Environmental Criteria and Risk Assessment Chinese Research Academy of Environmental Sciences 100012 Beijing China; ^3^ Beijing Institute of Nanoenergy and Nanosystems Chinese Academy of Sciences 100083 Beijing China; ^4^ MOE Key Laboratory of New Processing Technology for Non‐ferrous Metals and Materials Guangxi Key Laboratory of Processing for Non‐ferrous Metals and Featured Materials Guangxi University 530004 Nanning China; ^5^ School of Materials Science Engineering Zhengzhou University 450001 Zhengzhou P. R. China

**Keywords:** CsPbBr_3_, heterojunction, piezoelectric nanogenerator, self‐polarization, Ti_3_C_2_T*_x_*

## Abstract

Piezoelectric nanogenerator (PENG) for practical application is constrained by low output and difficult polarization. In this work, a kind of flexible PENG with high output and self‐polarization is fabricated by constructing CsPbBr_3_–Ti_3_C_2_T*_x_* heterojunctions in PVDF fiber. The polarized charges rapidly migrate to the electrodes from the Ti_3_C_2_T*_x_* nanosheets by forming heterojunctions, achieving the maximum utilization of polarized charges and leading to enhanced piezoelectric output macroscopically. Optimally, PVDF/4wt%CsPbBr_3_/0.6wt%Ti_3_C_2_T*_x_*‐PENG exhibits an excellent voltage output of 160 V under self‐polarization conditions, which is higher than other self‐polarized PENG previously. Further, the working principle and self‐polarization mechanism are uncovered by calculating the interfacial charge and electric field using first‐principles calculation. In addition, PVDF/4wt%CsPbBr_3_/0.6wt%Ti_3_C_2_T*_x_*‐PENG exhibits better water and thermal stability attributed to the protection of PVDF. It is also evaluated in practice by harvesting the energy from human palm taps and successfully lighting up 150 LEDs and an electronic watch. This work presents a new idea of design for high‐performance self‐polarization PENG.

## Graphical abstract

A flexible piezoelectric nanogenerator (PENG) with high output and self‐polarization is fabricated by constructing CsPbBr_3_–Ti_3_C_2_T*_x_* heterojunctions in PVDF fiber. The polarized charges rapidly migrate from the Ti_3_C_2_T*_x_* nanosheets to the electrodes by forming heterojunctions, achieving the maximum utilization of polarized charges and leading to enhanced piezoelectric output macroscopically. The PVDF/4 wt%CsPbBr_3_/0.6 wt% Ti_3_C_2_T*_x_*‐PENG exhibits an excellent voltage output of 160 V.

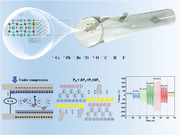

## 1 Introduction

The use of mechanical motion as a natural source of power is gradually replacing battery or electrical power in driving smart electronics. In recent years, piezoelectric nanogenerator (PENG) based on piezoelectric materials has gained tremendous attention for its remarkable ability to convert mechanical energy into electrical energy.^[^ ^1^ ^]^ Lead halide perovskites (ABX_3_, A = methyl ammonium, formamidinium (FA, Cs; B = Pb; X = Cl, Br, and I) have become a research hotspot as not only optoelectronics but also PENG due to its unique optical properties and asymmetry of crystal structure.^[^ ^2^ ^]^ The first lead halide perovskite employed in the fabrication of PENG is MAPbI_3_, which exhibits output voltage and current density of 2.7 V and 140 nA cm^−2^, respectively.^[^ ^3^ ^]^ Later, FAPbBr_3_‐based PENG is developed with output voltage and current density of 8.5 V and 3.8 µA cm^−2^, respectively.^[^ ^4^ ^]^ Considering the stability of perovskites as well as flexible applications, composites of piezoelectric polymers and perovskites are gradually being mushroomed. Polyvinylidene fluoride (PVDF) and its copolymers are the most widely used in PENG such as PVDF/MAPbI_3_,^[^ ^5^ ^]^ PVDF/MAPbBr_3_,^[^ ^6^ ^]^ PVDF/FAPbBr_3_,^[^ ^7^ ^]^ PVDF/MAPbI_3_,^[^ ^8^ ^]^ PVDF/CsPbBr_3_,^[^ ^9^ ^]^ and PVDF/CsPbI_3_,^[^ ^10^ ^]^ because of their relatively high piezoelectric coefficients.^[^ ^11^ ^]^ However, the dipoles of composite materials are generally in a random arrangement, which obviously limits the improvement of piezoelectric properties.^[^ ^12^ ^]^ In addition, the buffering effect of elastic polymer PVDF causes the composite material to deform unevenly, creating a piezoelectric potential difference that makes a certain amount of polarized charge to be trapped in the material without contributing to the output current.^[^ ^13^ ^]^ These reasons lead to the unsatisfactory piezoelectric properties of composites.

At present, there are three strategies to enhance the dipole orientation toward outstanding piezoelectric performance. The first approach is to induce dipole alignment of piezoelectric materials by an electric poling process.^[^ ^14^ ^]^ However, materials with low breakdown voltages and large coercivity voltages are difficult to be polarized. In addition, the electric polarization of piezoelectric materials usually requires a long time of treatment under high temperatures and strong voltage, resulting in wasted energy and higher manufacturing cost.^[^ ^15^ ^]^ Meanwhile, the removal of the external electric field and heating condition causes depolarization of the material, which damages the stability of piezoelectric properties.^[^ ^16^ ^]^ The second approach is to control the orientation of materials (called texturing).^[^ ^17^ ^]^ However, the special template and complex synthesis process of texturing cause it to be effective for 1D and 2D materials but not for granular materials.^[^ ^18^ ^]^ The third approach is the self‐polarization effect by introducing interfacial polarization between different components.^[^ ^19^ ^]^ By comparison, the self‐polarization method is more suitable for extensive industrial applications due to its low cost and simple synthesis process. Sultana et al. prepared PVDF/MAPbBr_3_ composites by electrospinning and found that the strong electric field and mechanical stretching of the electrospinning process caused in situ polarization of PVDF.^[^ ^6^ ^]^ According to our group's previous work, PVDF/CsPbBr_3_ composites can obtain high piezoelectric output (*V* = 33 V) without external electric field polarization.^[^ ^9c^ ^]^ Regrettably, the performance of piezoelectric composites obtained by this method is still far from electric poling.^[^ ^9a^ ^]^

Synthesizing heterojunctions with a strong built‐in electric field can effectively transfer electrons which is an effective way to relieve trapped polarized charge.^[^ ^20^ ^]^ MXene, as a promising energy storage material, has attracted great interest due to its unique features such as excellent electronic conductivity, obvious security capability, environmental benignity, and excellent biocompatibility.^[^ ^21^ ^]^ Liu et al. synthesized the MXene/COF/Cu_2_O heterojunction for photocatalytic sterilization.^[^ ^22^ ^]^ Efficient photocatalysis is achieved due to the strong built‐in electric field generated at the heterojunction interface promoting rapid carrier migration. The as‐constructed MoS_2_@MXene@D‐TiO_2_ heterostructure in sodium‐ion batteries delivers admirable high‐rate reversible capacity due to the built‐in electric field between the non‐homogeneous phases that promotes the high Na^+^ transportation.^[^ ^23^ ^]^ Meanwhile, Agresti et al. suggested that the formation of heterojunctions between MXene and perovskite could regulate the arrangement of dipole moments.^[^ ^24^ ^]^ Therefore, the construction of MXene heterojunction with perovskite is expected to improve both the arrangement of dipole moments and the utilization of polarized charges.

Herein, CsPbBr_3_–Ti_3_C_2_T*_x_* heterojunctions are first constructed in PVDF fibers, providing a path for the transfer of polarized charges inside the film and macroscopically enhancing the output performance of the devices. Meanwhile, the PVDF/CsPbBr_3_/Ti_3_C_2_T*_x_* composite achieved self‐polarization without an additional electric field. Further, the mechanism of self‐polarization is explained by analyzing the interfacial charge and electric field using first‐principles calculation. Besides, the piezoelectric outputs of PENG under experimental and practical conditions are also investigated systematically.

## 2 Results and Discussion

### 2.1 Preparation and Characterization of PVDF/CsPbBr_3_/Ti_3_C_2_T*_x_* Composite Fiber

**Figure 1:**
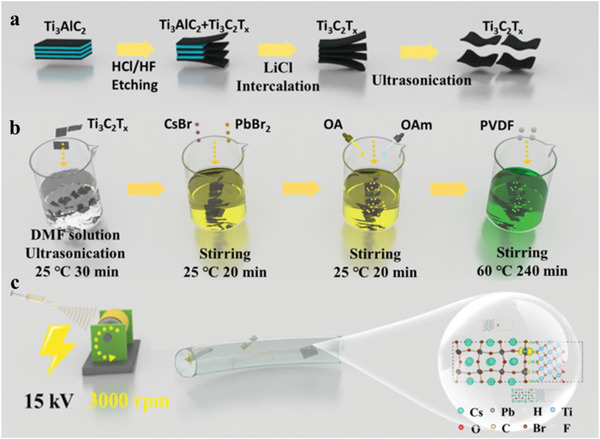
a) Preparation process of Ti_3_C_2_T*_x_* nanosheets. b) The synthesis process of composite precursor. c) Schematic illustration of the electrospinning system.

The PVDF/CsPbBr_3_/Ti_3_C_2_T*_x_* composite fibers were fabricated through the electrospinning method, as schematically illustrated in **Figure** **1**. First, Ti_3_C_2_T*_x_* nanosheets were synthesized by HF/HCl etching and LiCl intercalation of Ti_3_AlC_2_^[^ ^25^ ^]^ (Figure 1a). Then, the electrospinning precursor was prepared by a simple mixing process, whereby Ti_3_C_2_T*_x_*, CsBr, PbBr_2_, oleylamine (OAm), oleic acid (OA), and PVDF were added to DMF solution and homogenized via stirring at 60 °C (Figure 1b). According to our previous studies, PVDF/4 wt% CsPbBr_3_ exhibits the best self‐polarization effect and piezoelectric properties.^[^ ^9c^ ^]^ Thus, the 4 wt% CsPbBr_3_ was used to construct heterojunction with different contents of Ti_3_C_2_T *_x_* (0.2, 0.4, 0.6, 0.8, and 1.0 wt%). Finally, the precursor was drawn under the high electric field to obtain fibers (Figure 1c). The detailed preparation process and basic characterization of Ti_3_C_2_T*_x_* are described in Part 1 (Figures S1–S4, Supporting Information) of the Supporting Information. In addition, the elaborated preparation process of PVDF/CsPbBr_3_/Ti_3_C_2_T*_x_* composite fiber is shown in Part 2 (Figures S5 and S6, Supporting Information) of the Supporting Information.

**Figure 2:**
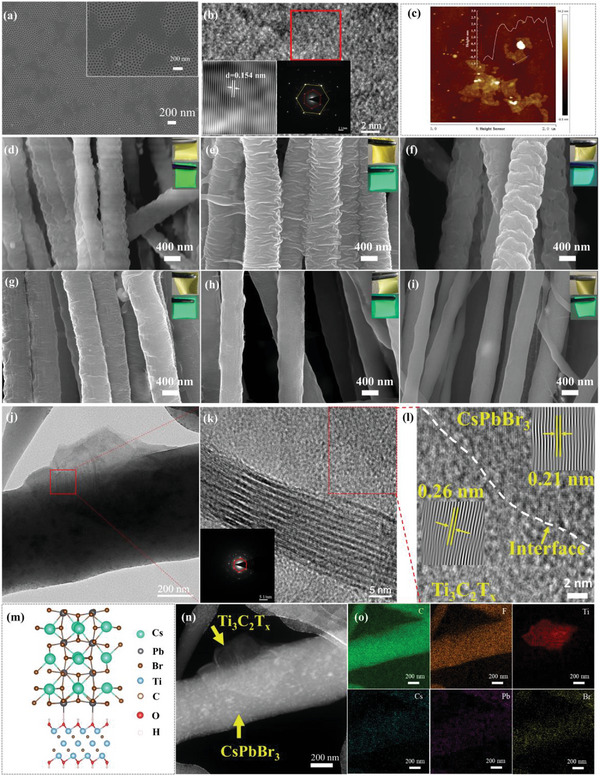
a) SEM, b) HRTEM and SAED, and c) AFM images of Ti_3_C_2_T*_x_*. d–i) SEM images of PVDF/CsPbBr_3_/Ti_3_C_2_T*_x_* (0 wt% Ti_3_C_2_T*_x_* (d), 0.2 wt% Ti_3_C_2_T*_x_* (e), 0.4 wt% Ti_3_C_2_T*_x_* (f), 0.6 wt% Ti_3_C_2_T*_x_* (g), 0.8 wt% Ti_3_C_2_T*_x_* (h), 1.0 wt% Ti_3_C_2_T*_x_* (i)). j) TEM image of PVDF/CsPbBr_3_/Ti_3_C_2_T*_x_* (0.6 wt% Ti_3_C_2_T*_x_*) fiber. k) HRTEM image and SAED of PVDF/CsPbBr_3_/Ti_3_C_2_T*_x_* (0.6 wt% Ti_3_C_2_T*_x_*) fiber. l) The interface of CsPbBr_3_–Ti_3_C_2_T*_x_* and corresponding Fourier transformation (Zoomed‐in view of (k)). m) The structure schematic diagram of the interface of CsPbBr_3_–Ti_3_C_2_T*_x_*. TEM images of PVDF/CsPbBr_3_/Ti_3_C_2_T*_x_* (0.6 wt% Ti_3_C_2_T*_x_*) fiber in the n) dark field and o) element mapping.

As shown in the scanning electron microscope (SEM) image, the lateral size of Ti_3_C_2_T*_x_* nanosheets is about 400 nm (**Figure** **2**a and Figure S3, Supporting Information) and the thickness is 2.4 nm (Figure 2c). The almost transparent transmission electron microscope (TEM) image (Figure S2e, Supporting Information) and the hexagonal arrangement of the atoms (Figure 2b) confirm the successful preparation of Ti_3_C_2_T*_x_* nanosheets. For X‐ray diffraction (XRD) pattern, the typical peak (104) of Ti_3_AlC_2_ disappears, and (002) shifts to a lower angle again confirming the successful preparation of ultrathin Ti_3_C_2_T*_x_* nanosheets (Figure S2j, Supporting Information). This exfoliation technique results in abundant surface termination with OH, F, O, and Cl (Figure S2k–p, Supporting Information), providing conditions for the formation of heterojunction.

SEM images of PVDF/CsPbBr_3_/Ti_3_C_2_T*_x_* composite fiber films are presented in Figure 2d–i. The fibers are uniform in diameter and high‐alignment in orientation. Optical images of fiber films under normal light and 365 nm ultra‐violet (UV) light are shown in the inset of Figure 2d–i. The green photoluminescence (PL) emission of the fiber implies the uniform growth of CsPbBr_3_ and Ti_3_C_2_T*_x_* in PVDF fibers. As shown in Figure S7, Supporting Information, the diameter of the fiber decreases with increasing Ti_3_C_2_T*_x_* content. In electrospinning process, droplets are charged to produce jets that stretch to form fibers after overcoming the surface tension of the liquid.^[^ ^26^ ^]^ Therefore, the size of the fiber is mainly influenced by the Coulomb force and surface tension. Viscosity tests for each component precursor are listed in Figure S8, Supporting Information. The viscosity of the precursor gradually increases with increasing Ti_3_C_2_T*_x_* content, which represents an increase in the surface tension of the precursor. Theoretically, the fiber diameter increases with the increase of surface tension. Here, the regular decrease in fiber diameter is due to the addition of Ti_3_C_2_T*_x_* improves the conductivity of the precursor, making the increase in Coulombic force greater than the effect of surface tension.

Figure 2j,n reveals the TEM image of PVDF/CsPbBr_3_/Ti_3_C_2_T*_x_* (0.6 wt% Ti_3_C_2_T*_x_*) fibers in dark and bright fields. It can be seen that CsPbBr_3_ is uniformly dispersed inside the fibers, while the Ti_3_C_2_T*_x_* is inserted into the fiber. High‐resolution transmission electron microscopy (HRTEM) images demonstrate that Ti_3_C_2_T*_x_* nanosheets retain the hexagonal crystal structure of the parent Ti_2_AlC phase (the insert of Figure 2k). The crystal plane spacing of 0.21 and 0.26 nm are corresponding to the (220) crystal plane of CsPbBr_3_^[^ ^27^ ^]^ and (100) crystal plane of Ti_3_C_2_T*_x_* ^[^ ^28^ ^]^ (Figure 2l). The white interface implies the formation of Ti_3_C_2_T*_x_*–CsPbBr_3_ heterojunction. The structure schematic diagram of the interface of CsPbBr_3_–Ti_3_C_2_T*_x_* is exhibited in Figure 2m, which will be discussed in detail in the calculation section. The corresponding element mapping also confirmed the homogeneous distribution of CsPbBr_3_ in the fibers and the presence of Ti_3_C_2_T*_x_* (Figure 2o).

**Figure 3:**
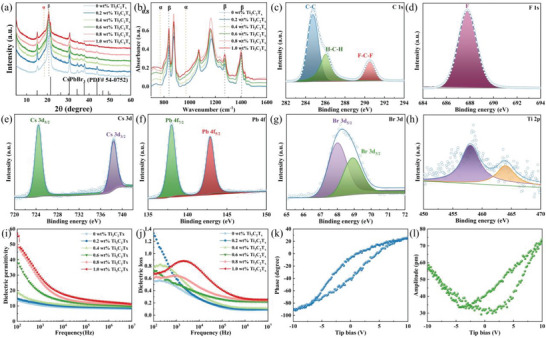
a) XRD and b) FTIR spectra of composite films. High‐resolution XPS spectra of PVDF/CsPbBr_3_/Ti_3_C_2_T*_x_* (0.6 wt% Ti_3_C_2_T*_x_*) composite film: c) C, d) F, e) Cs, f) Pb, g) Br, and h) Ti. i) Dielectric permittivity and j) dielectric loss of composite films. k) Phase and l) amplitude hysteresis loop of PVDF/CsPbBr_3_/Ti_3_C_2_T*_x_* (0.6 wt% Ti_3_C_2_T*_x_*) fiber.

XRD spectra of composite films are revealed in **Figure** **3**a. The two peaks prominent at 18.3° and 20.4° correspond to the *α* phase and *β* phase of PVDF. The peaks arise at 15.1°, 21.5°, 30.6°, 34.4°, 37.8°, and 43.9° correspond to the (100), (110), (200), (210), (211), and (220) crystal plane of CsPbBr_3_(PDF# 54–0752), confirming the formation of CsPbBr_3_ nanoparticles within the polymer fiber. However, due to the low loading weight percent and uniform dispersion of Ti_3_C_2_T*_x_*, the peak representing Ti_3_C_2_T*_x_* did not appear in XRD spectra. The crystalline forms of composite films are further explored by the fourier transform infrared spectroscopy (FTIR) as shown in Figure 3b. The calculation method of *β* phase content is exposed in the Supporting Information. With the addition of 4 wt% CsPbBr_3_, the *β* phase content increased from 78.7% to 94.6% (Figure S9, Supporting Information), due to the formation of a built‐in electric field by the addition of CsPbBr_3_ nanoparticles. The addition of Ti_3_C_2_T*_x_* brings the *β* phase content to a maximum of 95.4% due to a further increase of the built‐in electric field, which is confirmed in the calculation section.

The surface composition and chemical states of PVDF/CsPbBr_3_/Ti_3_C_2_T*_x_* (0.6 wt% Ti_3_C_2_T*_x_*) are investigated by X‐ray photoelectron spectroscopy (XPS). Figure S10, Supporting Information, unveils the full XPS spectrum of composite film, where the signals related to C, F, Cs, Pb, Br, and Ti. The C1s peak exhibit three components at 284.8, 286.5, and 291.0 eV corresponding to C—C, H—C—H, and F—C—F bond of PVDF, respectively (Figure 3c).^[^ ^29^ ^]^ F1s has a single peak at 688.1 eV, which is consistent with the organic fluorine (Figure 3d). Cs 3d_3/2_ and 3d_5/2_ peaks are observed at 738.2 and 724.3 eV (Figure 3e), consistent with the results of the Cs^+^ state. Peaks of Pb 4f_5/2_ and 4f_7/2_ appear at 146.8 and 141.9 eV (Figure 3f), representing the Pb^2+^ cations.^[^ ^30^ ^]^ The Br 3d_3/2_ and 3d_5/2_ have the binding energy of 69.3 and 68.2 eV, with energy differences of 1 eV (Figure 3g), corresponding to Br^−^ state. The peaks occur at 457.2 and 463.1 eV, corresponding to Ti—C bond of Ti_3_C_2_T*_x_* (Figure 3h), which again verifies the presence of Ti_3_C_2_T*_x_* in the fiber. The high‐resolution XPS spectra of PVDF, PVDF/CSPbBr_3_ and PVDF/CsPbBr_3_/Ti_3_C_2_T*_x_* are compared in Figure S11, Supporting Information. The interactions of H—C—H and F—C—F weaken with the addition of Ti_3_C_2_T*_x_*, which is the result of the enhanced interaction of the end groups F and H with Ti_3_C_2_T*_x_*. The peak of Pb 4f is shifted to the left by 4 eV after addition of Ti_3_C_2_T*_x_*, indicating a strong interaction between the Ti_3_C_2_T*_x_* and under‐coordinated Pb atoms, formatting the heterojunctions.

The performance of PENG is directly dependent on its piezoelectric constant (*d*_33_), which is proportional to the dielectric permittivity and the remnant polarization.^[^ ^31^ ^]^ Here, the dielectric permittivity and dielectric loss of composites are measured in Figure 3i,j. The dielectric permittivity of composites increases gradually from 14 for 0 wt% Ti_3_C_2_T*_x_* to the highest value of 58 for 1.0 wt% Ti_3_C_2_T*_x_* at the frequency of 100 Hz. The addition of Ti_3_C_2_T*_x_* increases the interfacial polarization, which is confirmed in the calculation section, leading to an increase in the dielectric coefficient.^[^ ^32^ ^]^ The dielectric loss is two orders of magnitude smaller than the dielectric constant. Due to the low‐frequency oscillation, more attention is paid to the data pattern after 1000 Hz. However, the dielectric loss increases with the increase of Ti_3_C_2_T*_x_* because the increase of Ti_3_C_2_T*_x_* forms a conducting path. The increase in dielectric loss is detrimental to the piezoelectric performance. Finally, a quasi‐static *d*_33_ meter was used to measure the *d*_33_ of PVDF/CsPbBr_3_/Ti_3_C_2_T*_x_* films as 36.1 pC/N. The detailed test method and procedure are shown in Figure S12, Supporting Information.

Further, the topography, amplitude, and phase images of piezoresponse force microscope (PFM) are shown in Figure S13, Supporting Information. The amplitude image clearly shows the piezoelectric response, while the phase image indicates the significant distribution of ferroelectric domains. Besides, the amplitude and phase response loops have been obtained by applying DC bias from −10 to +10 V. The phase response loop indicates the polarization change under electric field (Figure 3k). The butterfly‐shaped amplitude loop indicates the electrostriction induced by the inverse piezoelectric effect (Figure 3l). The two transition points near the bottom of the loop represent the dipole switching behavior. The slight offset near the center of the loop reveals that there exists a built‐in field within the film generated by the spontaneous polarization.

### 2.2 Performance of PENG Based on PVDF/CsPbBr_3_/Ti_3_C_2_T*_x_* Composite Fiber

**Figure 4:**
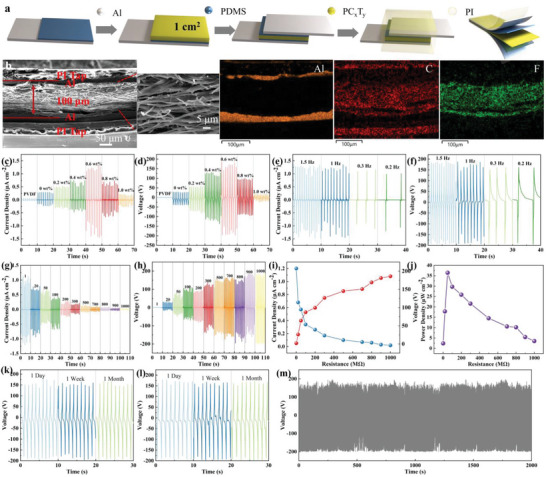
a) The fabrication procedure of PENG. b) The cross‐section SEM image of the device and corresponding element mapping. The c) *I*_sc_ density and d) *V*_oc_ of PENG based on composite. The e) *I*_sc_ density and f) *V*_oc_ of PENG based on PVDF/CsPbBr_3_/Ti_3_C_2_T*_x_* (0.6 wt% Ti_3_C_2_T*_x_*) under different frequencies. The g) *I*_sc_ density and h) *V*_oc_ of PENG based on PVDF/CsPbBr_3_/Ti_3_C_2_T*_x_* under different external load resistance from 1 MΩ to 1 GΩ. i,j) The tendency of *I*_sc_ density, *V*_oc_, and the power density of PVDF/CsPbBr_3_/Ti_3_C_2_T*_x_* under different external load resistance from 1 MΩ to 1 GΩ. The output of k) PVDF/CsPbBr_3_/Ti_3_C_2_T*_x_* under 100 °C for different times and l) soaking in water for different times. m) Output recorded over time for continuous 2000 cycles.

To investigate the piezoelectric properties of composite films, the top–bottom configuration of PENG is prepared, where polydimethylsiloxane (PDMS) is a surface adhesion and passivation layer. The detailed fabrication procedure of PENG is illustrated in **Figure** **4**a. Figure 4b exhibits the cross‐section SEM image and corresponding element mapping. It can be seen that the thickness of the film is about 100 µm and it is closely connected to the Al foil electrode, avoiding the influence of triboelectricity. The effective device size of the film is 1 × 1 cm^2^.

The piezoelectric output of short‐circuit current (*I*_sc_) density and open‐circuit voltage (*V*_oc_) of composite are measured under palm tap (Figure 4c,d). The tapping force is about 25 N (Figure S14, Supporting Information) and the frequency is 1 Hz. The *I*_sc_ density increased from 0.3 to 1.3 µA cm^−2^ as Ti_3_C_2_T*_x_* mass fraction increased from 0 to 0.6 wt%. The *I*_sc_ density of 0.6 wt% Ti_3_C_2_T*_x_* is about 4.3 times larger than 0 wt% Ti_3_C_2_T*_x_*. Similarly, as the Ti_3_C_2_T*_x_* increased from 0 to 0.6 wt%, *V*_oc_ increased from 33 to 160 V and then decreased from 160 to 15 V with a further increase in Ti_3_C_2_T*_x_*. The best performance was obtained at 0.6 wt% Ti_3_C_2_T*_x_* addition caused by the most appropriate dielectric constant and dielectric loss values. The photograph of the test system is shown in Figure S15, Supporting Information. Thus PVDF/CsPbBr_3_/Ti_3_C_2_T*_x_* (0.6 wt% Ti_3_C_2_T*_x_*) was selected for subsequent tests. The performance of PVDF/CsPbBr_3_/Ti_3_C_2_T*_x_* at different frequencies was examined in Figure 4e,f. The *I*_sc_ and *V*_oc_ remain stable in the range of 0.2–1.5 Hz, indicating that they can perform energy harvesting and conversion tasks in a wide frequency range. **Table** **1** compares the piezoelectric performance based on some self‐polarized PENG. Compared to other self‐polarized PENG devices, the PVDF/CsPbBr_3_/Ti_3_C_2_T*_x_* has a significant output voltage. A switching‐polarity test was performed as shown in Figure S16, Supporting Information. It verifies that the output signal comes from the PENG and not from the instrument.

**Table 1:** Comparison of piezoelectric performance based on some self‐polarized PENGs

Composite	Preparation method	Output voltage [V]	Applied force	Active area [cm^2^]	Ref.
PVDF/Gly‐MoS_2_	Casted	8.2	50 KPa	1 × 2	[33]
PVDF/FAPbBr_3_	Casted	20	0.5 MPa	1.2 × 1.4	[7]
PVDF/PZT	3D printing	60	255 KPa	1 × 1	[34]
PVDF‐TrFE/CNT	Electrospinning	0.4	—	2.1	[19c]
PVDF/h‐BN	Electrospinning	8.3	—	1 × 1	[35]
PVDF/MAPbI_3_	Casted	1.8	Finger tap	1 × 1	[5]
PVDF/PtNPs	Casted	18	Finger tap	0.3	[36]
PVDF/MAPbBr	Electrospinning	5	Finger tap	2.4 × 1.5	[6]
PVDF/CsPbBr_3_	Casted	120	Finger tap	—	[37]
PVDF/CsPbBr_3_/Ti_3_C_2_T*_x_*	Electrospinning	160	Palm tap	1 × 1	This work

Figure 4g–j displays the *I*_sc_ and *V*_oc_ of PVDF/CsPbBr_3_/Ti_3_C_2_T*_x_* with external resistance load. The external load range is from 1 MΩ to 1GΩ, the *I*_sc_ density drops from 1 µA cm^−2^ to 0.2 nA cm^−2^ and *V*_oc_ rises from 2 to 160 V. The power density of PVDF/CsPbBr_3_/Ti_3_C_2_T*_x_* reaches the maximum value of 36.4 µW cm^−2^ at an external contact resistance of 100 MΩ. Besides, thermal and water stability of the device was investigated by recording the output at 100 °C and immersed water at different times. The *V*_oc_ of PVDF/CsPbBr_3_/Ti_3_C_2_T*_x_* decreases from 160 to 150 V when held at 100 °C for 1 day to 1 month (Figure 4k). Further, the *V*_oc_ of PVDF/CsPbBr_3_/Ti_3_C_2_T*_x_* remained stable after immersing the device in water for 1 day, 1 week, and 1 month (Figure 4l). Finally, a continuous stability test is conducted to investigate the lifetime of PVDF/CsPbBr_3_/Ti_3_C_2_T*_x_*. After 2000 cycles, it still maintains a large output with *V*_oc_ of 160 V (Figure 4m).

### 2.3 Mechanism Analysis

First, to explain the observed piezoelectric properties and reveal the basic fundamental of heterojunction‐enhanced self‐polarization, it is crucial to understand the interactions between substances in composites. Here, the electron distribution and electric field magnitude of Ti_3_C_2_T*_x_*, CsPbBr_3_, and PVDF interface are calculated separately using first‐principles calculation. The PVDF–CsPbBr_3_ interfacial interactions have been studied in previous papers.^[^ ^9c^ ^]^ Here, we focus on the interfacial interactions between Ti_3_C_2_T*_x_*–CsPbBr_3_ and Ti_3_C_2_T*_x_*–PVDF. To quantify the stability of the interfaces, the binding energy (*E*_b_) is first calculated by Equation (1):

(1) $$E_{b}=\;\left(E_{\mathrm{AB}}-E_{A}-E_{B}\right)/S$$

where *E*_AB_, *E*_A_, and *E*_B_ are the energy of interfaces. *S* is the surface area. The calculated *E*_b_ of Ti_3_C_2_T*_x_*–CsBr, Ti_3_C_2_T*_x_*–PbBr, and Ti_3_C_2_T*_x_*–PVDF are −0.0189, −0.0707, and −0.0093 eV Å^−2^, respectively. The negative *E*_b_ suggests that the interfaces could form in the experiments in the view of thermodynamics. Nevertheless, the more negative *E*_b_ of Ti_3_C_2_T*_x_*–PbBr indicates that it is more stable in the experiments.

**Figure 5:**
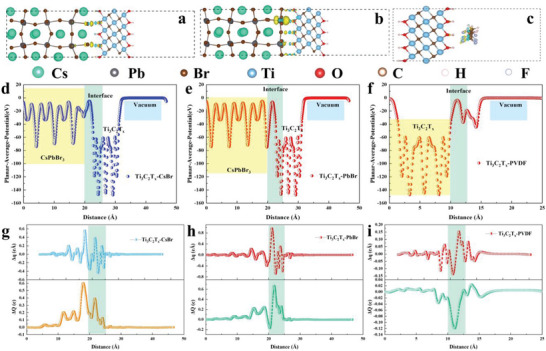
Interface induced charge density isosurface. a) Ti_3_C_2_T*_x_*‐CsBr, b) Ti_3_C_2_T*_x_*‐PbBr, and c) Ti_3_C_2_T*_x_*‐PVDF. Planar averaged electrostatic potential. d) Ti_3_C_2_T*_x_*‐CsBr, e) Ti_3_C_2_T*_x_*‐PbBr, and f) Ti_3_C_2_T*_x_*‐PVDF. Charge density difference and charge displacement curve of g) Ti_3_C_2_T*_x_*‐CsBr, h) Ti_3_C_2_T*_x_*‐PbBr, and i) Ti_3_C_2_T*_x_*‐PVDF.

The charge density isosurfaces of the charge redistribution upon formation of Ti_3_C_2_T*_x_*–CsBr, Ti_3_C_2_T*_x_*–PbBr, and Ti_3_C_2_T*_x_*–PVDF are calculated and displayed in **Figure** **5**a–c. The cyan and yellow isosurfaces set as 0.0005 e a_0_^−3^ reflect the charge depletion and accumulation region, respectively. There is a large amount of charge transfer and exchange between Ti_3_C_2_T*_x_* and PbBr. It can be seen that the charge exchange is mainly concentrated between the Pb and O atoms.

(2) $$\Delta q\;=\int_{-\infty }^{+\infty }\int_{-\infty }^{+\infty }\left(\rho_{\mathrm{AB}}-\rho_{A}-\rho_{B}\right)dxdy\;$$

where *ρ*_AB_, *ρ*_A_, and *ρ*_B_ are the charge density of interfaces AB, A, and B, respectively. Based on ∆*q*, the charge displacement curve is calculated by Equation (3):

(3) $$\Delta Q\;=\int_{-\infty }^{z}\Delta \rho dz\;$$

The positive and negative gradients of *∆Q* in the interfacial region represent charge accumulation and depletion. The planar average electrostatic potential and the local potential at the interface are shown in Figure 5d–f. *∆Q* of CsPbBr_3_–Ti_3_C_2_T*_x_* and PVDF–Ti_3_C_2_T*_x_* heterojunction is shown in Figure 5g–i. The charge information and electric field at the interface of CsPbBr_3_ and PVDF are shown in Figure S17, Supporting Information. The comparison reveals that the interfacial charge aggregation of Ti_3_C_2_T*_x_*–PbBr was at most 0.7 e Å^−1^, causing a strong electric field at the interface, which provides the necessary conditions for the self‐polarization of the composite and the movement of the polarized charge. Meanwhile, the strong interaction between Ti_3_C_2_T*_x_* and the undercoordinated Pb atom provides conditions for the formation of heterojunctions, which has been confirmed by the results of XPS and TEM. As a result, the heterojunction significantly enhances the self‐polarization of the composite and makes the dipole arrangement more orderly, thus enhancing the piezoelectric properties of the composite.

**Figure 6:**
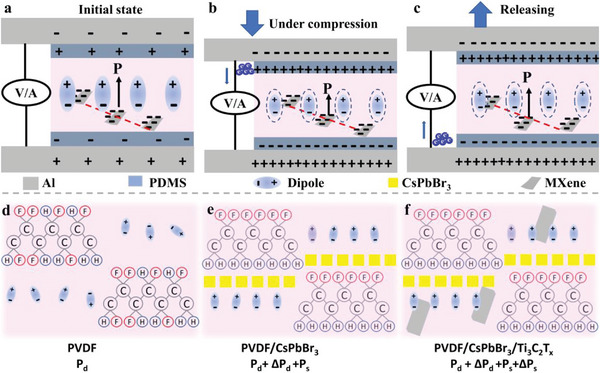
a–c) The working mechanism of PVDF/CsPbBr_3_/Ti_3_C_2_T*_x_*‐PENG. d–f) Schematic illustration of the contribution of the sample to the polarization.

Second, the origin of the direct piezoelectric effect stems from the behavior of the surface charge as the material is subjected to stress that changes the polarization level.^[^ ^38^ ^]^ PVDF has a force buffering effect that retains a certain amount of polarized charge in the material thus weakening the piezoelectric output of the material. Zhou et al. created a multi‐layered PENG with a 3D interdigitated electrode that effectively exported the internal polarized charge, resulting in a significant increase in output current.^[^ ^39^ ^]^ Therefore, we construct CsPbBr_3_–Ti_3_C_2_T*_x_* heterojunctions in PVDF fibers to provide a path for the transfer of polarized charges inside the film. The band structure of PVDF/CsPbBr_3_/Ti_3_C_2_T*_x_* is shown in Figure S18, Supporting Information. The band gap of heterostructure is zero, which provides sites and channels for the aggregation and transfer of polarized charges. The working mechanism of composite is displayed in **Figure** **6**. For PVDF/CsPbBr_3_/Ti_3_C_2_T*_x_*‐PENG, the electric dipoles of the composite have relatively uniform orientation under the effect of self‐polarization of the built‐in electric field (Figure 6a). Under compression to the PENG, the presence of self‐polarization with deformation dipoles leads to the generation of polarization charges on the material surface, which are attracted to the charged surfaces of material and accumulate to form a piezoelectric potential. The external free charges migrate to the electrodes and accumulate to balance the piezoelectric potential. The heterogeneous interface between CsPbBr_3_ and Ti_3_C_2_T*_x_* can be regarded as a Schottky barrier,^[^ ^40^ ^]^ which dominates the transport process of free charges. As a result, the polarization charges are first trapped and aggregated on the Ti_3_C_2_T*_x_* nanosheets and migrate rapidly through Ti_3_C_2_T*_x_* to the electrode's surface, leading the more polarization charges on the electrode's surface (Figure 6b). When the external stress is released, the accumulated charges flow back in the opposite direction (Figure 6c).

Finally, under the strong electric field and mechanical stretching of electrospinning, PVDF with 78% content of *β* phase has a certain net dipolar (*P*_d_) polarization (Figure 6d). CsPbBr_3_ acts as a nucleating agent to increase the *β* phase of PVDF to 94%, leading to a high extra polarization (Δ*P*_d_). In addition, CsPbBr_3_ also induces space charge polarization (*P*_s_) at the interface of the polymer matrix (Figure 6e). When Ti_3_C_2_T*_x_* was introduced, both the *β* phase content and space charge polarization are further enhanced. Accordingly, the presence of both fillers may ultimately result in considerable changes in polarization as expressed with the summation of each contribution, *P*_d_ + Δ*P*_d_ + *P*_s_ + Δ*P*_s_ (Figure 6f).

### 2.4 Practical Application of PENG

**Figure 7:**
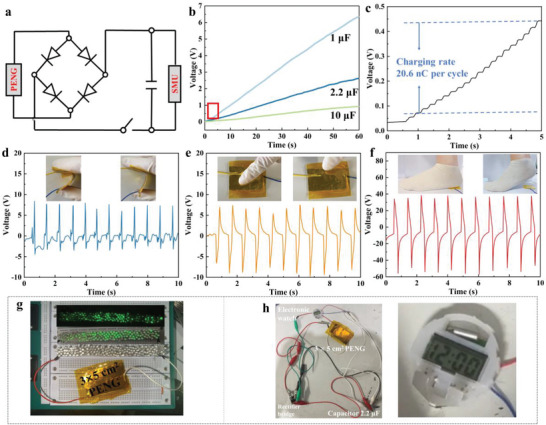
a) The circuit diagram of a capacitor being charged. b) Charging curves of capacitors with different capacitance values. c) Zoomed‐in view of the charging curve of 1 µF from 0 to 5 s. The output of PENG under d) finger bending, e) finger tapping, and f) foot stepping. g) Light up LEDs and h) commercial electronic watch.

To test the charging capability of the device, the AC signal is converted into a DC signal by a rectifier bridge. **Figure** **7**a depicts the circuit diagram of a capacitor being charged. The recorded energy‐storing process is presented in Figure 7b. The capacitors with different capacitances of 1, 2.2, and 10 µF are charged to 6.3, 2.6, and 0.9 V in 60 s. Figure 7c is the zoomed‐in view of the red square marked in Figure 7b from 1 to 5 s. The capacitor (1 µF) is charged from 0.44 to 0.07 V in 18 working cycles, the corresponding charging rate can be calculated as 20.6 nC per cycle, reaffirming the considerable piezoelectric outputs.

The piezoelectric response of several movements such as finger bending, finger tapping, and foot stepping were investigated to explore the collection of mechanical energy of human movement by PVDF/CsPbBr_3_/Ti_3_C_2_T*_x_*‐PENG (Figure 7d–f). It can be seen that the PENG has a good piezoelectric response to the mechanical energy of human movement. Meanwhile, the response signal of pressure is more prominent compared with bending. Without any storage device, the PVDF/CsPbBr_3_/Ti_3_C_2_T*_x_*‐PENG lit 150 commercial green LEDs (Figure 7g and Video S1, Supporting Information). In addition, a commercial electronic meter is successfully lit using a 2.2 µF capacitor voltage regulator (Figure 7h and Video S2, Supporting Information).

## 3 Conclusion

High output and self‐polarized PVDF/4 wt% CsPbBr_3_/0.6 wt% Ti_3_C_2_T*_x_*‐PENG is prepared by constructing CsPbBr_3_–Ti_3_C_2_T*_x_* heterojunction. On the one hand, PVDF/CsPbBr_3_/Ti_3_C_2_T*_x_* exhibits high polarization of *P*_d_ + Δ*P*_d_ + *P*_s_+ Δ*P*_s_ due to high *β* phase content and strong space charge polarization. On the other hand, the CsPbBr_3_–Ti_3_C_2_T*_x_* heterojunction provides a channel to accumulate and transfer polarization charges, making full utilize the polarized charges inside of film, leading to the macroscopic enhancement of its piezoelectric output. Meanwhile, the strong interfacial electric field induced by the interfacial interaction of CsPbBr_3_–Ti_3_C_2_T*_x_* enables the composites to achieve self‐polarization, making it possible to produce energy‐efficient harvesters with low energy consumption. PVDF/4 wt% CsPbBr_3_/0.6 wt% Ti_3_C_2_T*_x_*‐PENG exhibits outstanding voltage output of 160 V, which is higher than other self‐polarization PENG previously. In addition, it exhibits better water and thermal stability attributed to the protection of PVDF. The outstanding output can charge a 1 µF capacitor from 0 to 6.3 V in 60 s. In application, PVDF/4 wt% CsPbBr_3_/0.6 wt% Ti_3_C_2_T*_x_*‐PENG can directly power commercial green 150 LEDs without external storage and an electronic meter using a 2.2 µF capacitor voltage regulator.

## 4 Experimental Section

### Preparation of Ti_3_C_2_T*_x_* Nanosheets

Ti_3_C_2_T*_x_* nanosheets were synthesized by selective etching of Al from Ti_3_AlC_2_ (particle size <30 µm) using HF/HCl etchant. The etching solution was prepared by mixing 6 mL deionized water (DI water), 12 mL hydrochloric acid (HCl, Aladdin, 37%), and 2 mL hydrofluoric acid (HF, Macklin, 40%). 1 g Ti_3_AlC_2_ was slowly added to the etchant solution for about 5 min and then stirred at 400 rpm for 24 h at 35 °C. The as‐obtained multilayer Ti_3_C_2_T*_x_* was washed with DI water and centrifuged at 3500 rpm (5 min per time) until pH ≥ 6. The precipitate was collected and redispersed into 20 mL DI water by shaking, and then added to a solution of 40 mL DI water containing 1 g LiCl and stirred at 400 rpm for 4 h at 35 °C. The obtained Ti_3_C_2_T*_x_* dispersions were washed by centrifugation at 8000 rpm using DI water until the pH exceeded 6. Further, the sediment was redispersed in 35 mL DMF solution to probe sonicated in a cold bath for 20 min (power: 600 w). The supernatant was collected by centrifugation at 3500 rpm for 1 h. For the quantitative analysis of Ti_3_C_2_T*_x_* content in DMF, 2 mL of the solution was dried under a vacuum at 60 °C.

### Fabrication of PVDF/CsPbBr_3_/Ti_3_C_2_T*_x_* Composite Nanogenerator

The composite nanofibers were prepared by the electrospinning method. Initially, the Ti_3_C_2_T*_x_* suspension (solvent was DMF) was added to the DMF solution and sonicated for 30 min to obtain a uniformly dispersed solution. Subsequently, CsBr and PbBr_2_ were added to the above solution and stirred for 20 min to dissolve it fully. The OAm and oleic acid (OA) were added and stirred magnetically for 20 min at 500 rpm. Finally, PVDF (molecular weight 1 200 000) was added to obtain the precursor solution by magnetically stirring at 60 °C for 4 h with a weight concentration of 14%. The prepared precursor was drawn into a syringe with a 0.5 mm inner diameter stainless‐steel needle for electrospinning. Electrospinning fibers were collected at a stainless‐steel rotating cartridge collector with a speed of 3000 rpm. The distance from the needle to the collector was 18 cm. The flow rate was maintained at 0.04 mm min^−1^. The applied voltage was set as 15 kV. Inside the electrospinning machine, the temperature was set to 30 °C, and the humidity was adjusted to 60%. Then, the electrospinning film was dried for 2 h in a normal oven at 80 °C. PENG was the top‐bottom–electrode configuration.

First, electrospinning fiber film was cut to the appropriate size (1 cm × 1 cm) as an active layer. Then the aluminum foil serves as the top and bottom electrodes bonded to both sides of the active layer via PDMS. Finally, it was encapsulated by polyimide (PI) tape.

### Characterization and Measurements

The surface morphology of the samples was characterized by a field emission scanning electron microscope on Gemini Sigma 300/VP. Microstructure of Ti_3_C_2_T*_x_* and PVDF/CsPbBr_3_ fiber was performed by TEM, selected area electron diffraction (SAED), and HRTEM on Tecnai G2 F30 S‐TWIN. Atomic Force Microscope (AFM) was utilized to test the morphology and thickness of Ti_3_C_2_T*_x_* nanosheets. Besides, the local piezoelectric properties were investigated with PFM based on an AFM system (Bruker Multimode 8). The dielectric properties were measured by a precision LCR meter (Agilent 4294A) at room temperature. The absorbance of Ti_3_C_2_T*_x_* in DMF solution was measured by a UV–vis spectrophotometer (Persee, TU‐1901). The viscosity of the precursor was obtained by a viscometer (DV2T‐LV). The phase structure was characterized using XRD on Ultima IV and FTIR on Nicolet IS10. The surface electronic states of PVDF/CsPbBr_3_ fiber film were measured by XPS using AXI ULTRA with a monochromatic Al K*α* source. Electrical analysis was accomplished by applying an external force on the PENG through a linear motor (Lin Mot, BSDLCA32‐012/LCC) and recorded by a source measure unit (Keithley Instruments). The quasi‐static *d*_33_ measuring instrument (ZJ‐3AN) was used to measure *d*_33_. The value of the palm tap force was measured by a pressure sensor (SIMBATOUCH‐SBT961M).

### Computational Details

The first‐principles calculation was performed in the framework of density functional theory (DFT) via Vienna ab‐initio simulation package code.^[^ ^41^ ^]^ The exchange–correlation functional was presented by the generalized gradient approximation in the form of Perdew–Burke–Ernzerhof.^[^ ^42^ ^]^ The core–electron interaction was treated by the projector‐augmented wave method.^[^ ^43^ ^]^ The long‐range weak van der Waals interactions were described by Grimme's DFT‐D3 dispersion correction.^[^ ^44^ ^]^ The cutoff energy was set as 460 eV. The convergency criteria of energy and Hellman–Feynman forces on each atom were set as 1 × 10^−4^ eV per atom and 0.05 eV Å^−1^, respectively. The Brillouin zone was sampled by a k‐point mesh 4 × 4 × 3. Based on the optimized CsPbBr_3_ crystal, the slab models with seven octahedron layers were constructed by slicing the CsPbBr_3_ crystal along (110) lattice plane. The slab terminated by Cs—Br and Pb—Br were both considered and a vacuum layer with the size of 20 Å was added along *z*‐axis to avoid interactions between mirror slabs. The dipole correction along *z*‐axis of slab was implemented to correct the errors introduced by the periodic boundary conditions. Considering the limited computational resource, a section of the PVDF was selected to investigate the interaction between CsPbBr_3_‐PVDF and Ti_3_C_2_T*_x_*‐PVDF.

## Conflict of Interest

The authors declare no conflict of interest.

## Supporting information

Supporting Information

Supplemental Video 1

Supplemental Video 2

## Data Availability

The data that support the findings of this study are available from the corresponding author upon reasonable request.
