# Supplementary material for: Heterojunction Engineering Enhanced Self‐Polarization of PVDF/CsPbBr3/Ti3C2T x Composite Fiber for Ultra‐High Voltage Piezoelectric Nanogenerator
Source: Adv Sci (Weinh). 2023 May 11;10(18):2300650. doi: 10.1002/advs.202300650 (PMC10288227; doi:10.1002/advs.202300650)
Supplement: Supplementary file 1 — Supporting Information [file ADVS-10-2300650-s003.pdf]

Heterojunction Engineering Enhanced Self-polarization of PVDF/$CsPbBr_3$/$Ti_3C_2T_x$ Composite Fiber for Ultra-high Voltage Piezoelectric Nanogenerator

You Xue [a], *Tao Yang* [a,*], *Yapeng Zheng* [a], *Kang Wang* [a], *Enhui Wang* [a], *Hongyang Wang* [b], *Laipan Zhu* [c], *Zhentao Du* [d], *Hailong Wang* [e], *Kuo-Chih Chou* [a], *Xinmei Hou* [a,*]

[a] Institute for Carbon Neutrality, University of Science and Technology Beijing. Beijing 100083, China

[b] State Key Laboratory of Environmental Criteria and Risk Assessment, Chinese Research Academy of Environmental Sciences, Beijing 100012, China

[c] Beijing Institute of Nanoenergy and Nanosystems, Chinese Academy of Sciences, Beijing 100083, China

[d] MOE Key Laboratory of New Processing Technology for Non-ferrous Metals and Materials, Guangxi Key Laboratory of Processing for Non-ferrous Metals and Featured Materials, Guangxi University, Nanning 530004, China

[e] School of Materials Science Engineering, Zhengzhou University, Zhengzhou City 450001, P. R. China

1. Preparation and properties of $Ti_3C_2T_x$ MXene nanosheets

$Ti_3C_2T_x$ nanosheets were synthesized by selective etching of Al from Ti_3AlC_2 (particle size < 30 μm) using HF/HCl etchant as *Gogotsi* reported.[1] The etching solution was prepared by mixing 6 mL deionized water (DI water), 12 mL hydrochloric acid (HCl, Aladdin, 37%), and 2 mL hydrofluoric acid (HF, Macklin, 40%). 1 g Ti_3AlC_2 was slowly added to the etchant solution for about 5 min and then stir at 400 rpm for 24 h at 35 °C. The as-obtained multilayer $Ti_3C_2T_x$ was washed with DI water and centrifuged at 3500 rpm (5 min/time) until pH ≥ 6 (as shown in **Figure S1**a). The precipitate was collected and redispersed into 20 mL DI water by shaking, and then added to a solution of 40 mL DI water containing 1 g LiCl and stirred at 400 rpm for 4 h at 35 °C. The obtained $Ti_3C_2T_x$ dispersions were washed by centrifugation at 8000 rpm using DI water until the pH exceeded 6. The volume of the precipitation after LiCl intercalation underwent significant expansion, suggesting the success of the intercalation (as shown in **Figure S1**b and c). **Figure S1**d and e show the photographs of aqueous monolayer $Ti_3C_2T_x$ solutions and laser irradiation, demonstrating the successful preparation of monolayer $Ti_3C_2T_x$. Further, the sediment was redispersed in 35 mL DMF solution to probe sonicated in a cold bath (−10 °C) for 20 min (power: 600 w). The supernatant was collected by centrifugation at 3500 rpm for 1h. For the quantitative analysis of $Ti_3C_2T_x$ content in DMF, 2 ml of the solution was dried under vacuum at 60 °C.

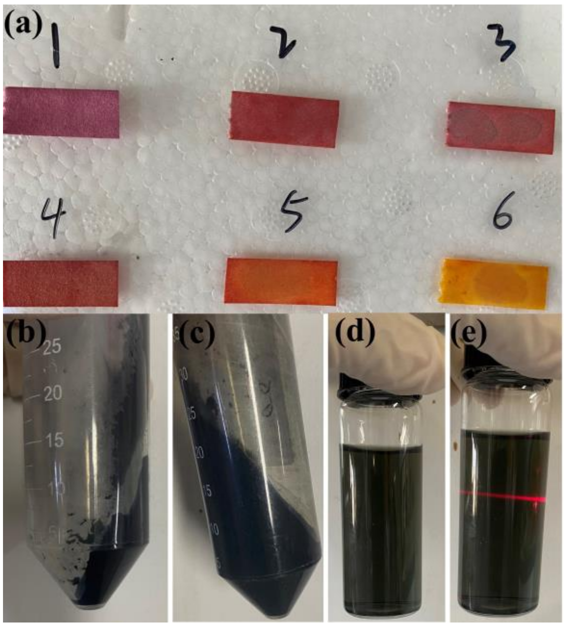

Figure S1 pH change of acid wash process after etching(a). The precipitation after HF/HCl etching and cleaning(b). The precipitation after LiCl intercalation(c). Monolayer $Ti_3C_2T_x$ water solution(d) and laser irradiation(e).

The surface morphology of Ti_3AlC_2, $Ti_3C_2T_x$, and intermediate products were characterized by a field emission scanning electron microscope (FE-SEM, Gemini Sigma 300/VP). The microscopy of bilayer and single $Ti_3C_2T_x$ nanosheets were study by transmission electron microscope (TEM, Tecnai G2 F30 S-TWIN). Atomic Force Microscope (AFM, Bruker Multimode 8) was utilized to test the morphology and thickness of $Ti_3C_2T_x$ nanosheets. The X-ray powder diffraction (XRD) patterns of the Ti_3AlC_2 phase and the $Ti_3C_2T_x$ nanosheets were obtained using a powder diffractometer (SMARTLAB(9)) equipped with a Cu Kα radiation (40 kV, 30 mA). The X-ray photoelectron spectroscopy (XPS) data on the $Ti_3C_2T_x$ nanosheets were acquired using Thermo ESCALAB 250XI equipped with Al Kα source (hν = 1486.6 eV) operating at 14.6 kW at a step of 0.1 eV.

The $Ti_3C_2T_x$ nanosheets were synthesized from Ti_3AlC_2 by selective etching of the Al using HF/HCl etchant. The fabrication process of $Ti_3C_2T_x$ nanosheets and the corresponding SEM images are illustrated in **Figure S2**a-d. The typical multilayered accordion-like morphology of $Ti_3C_2T_x$ is prepared by a general method for etching Ti_3AlC_2 with HF/HCl. Then, the multilayer $Ti_3C_2T_x$ was intercalated with the excellent intercalant LiCl to obtain the monolayer of $Ti_3C_2T_x$ with the size about 2-7 μm. The monolayer $Ti_3C_2T_x$ nanosheets with a size of about 400 nm were obtained by ultrasonic treatment for 30 min. The corresponding size statistics are shown in **Figure S3**. TEM image of single and bilayer $Ti_3C_2T_x$ nanosheets are shown in **Figure S2**e It can be seen that the nanoflakes are almost transparent. Several layers of nanosheets can be found in the center of the image (**Figure S2**f). The layer spacing is about 1.289 nm. Further, the HRTEM image of the monolayer $Ti_3C_2T_x$ is shown in **Figure S2**g. The lattice spacing obtained after Fourier transformation is 0.154 nm, which coincides with the ($\bar{2}110$) crystal plane of $Ti_3C_2T_x$.[2] The calculated interplanar crystal spacing are displayed in **Figure S4**. Meanwhile, the SAED pattern (**Figure S2**h) shows a hexagonal arrangement of atoms, in agreement with literature[3]. To further evaluate the etching status, the XRD patterns of the prepared nanosheets were compared with those of origin MAX. The AFM plot shows that the thickness of MXene is about 2.4 nm, indicating the successful preparation of monolayer-like $Ti_3C_2T_x$ nanosheets (**Figure S2**i). **Figure S2**j shows that the typical peak (104) of Ti_3AlC_2 disappeared after the treatment, indicating that the Al layers are totally etched away [4]. Meanwhile, the diffraction peaks of (002) shifted to a lower angle, demonstrating the successful preparation of ultrathin $Ti_3C_2T_x$ nanosheets.

XPS was used to further characterize the chemical state of the elemental composition of $Ti_3C_2T_x$ nanosheets. **Figure S2**k displays the full XPS spectrum, which includes Ti, C, O, F, Cl, where elements O, F, and Cl are present as surface terminators and caused by HF/HCl etching. In **Figure S2**l, the Ti 2p presents the Ti-C(I), Ti-C(II), and Ti-Cl bond with binding energies of 455.32(461.30) eV, 456.09(462.09) eV, and 459.39(465.40) eV. The C 1s peaks (**Figure S2**m) at 282.17 eV, 284.73 eV, and 286.73 eV are corresponding to C-Ti-T_x, C-C, and C-O bond,[5] respectively. The O 1s (**Figure S2**n**)** components are located at 531.04 eV, 531.84 eV, and 533.37 eV corresponding to C-Ti-O_x, C-Ti-$(OH)_x$, and adsorbed or interlayer water,[6] respectively. The Ti-F signal with binding energies of 685.32 eV is shown in **Figure S2**o. Presence of the Ti-Cl bond is also supported by identification of its corresponding $2p_{3/2}$ and $2p_{1/2}$ peak at 199.48 eV and 201.1 eV in the Cl 2p spectrum (**Figure S2**p).[7]

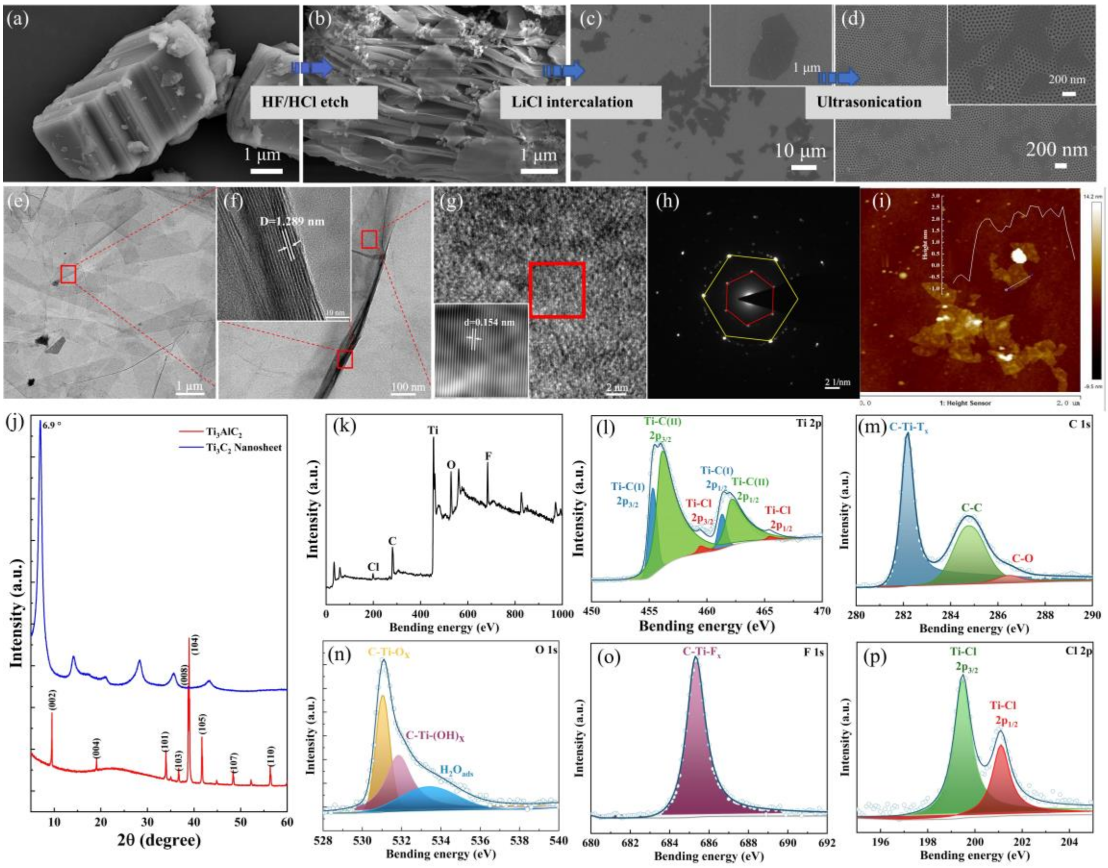

Figure S2 SEM images of primitive Ti_3AlC_2(a), HF/HCl etching(b), LiCl intercalation(c), and ultrasonication(d). TEM images of $Ti_3C_2T_x$ after LiCl intercalation(e). Single and bilayer $Ti_3C_2T_x$ sheets(f). HRTEM image of $Ti_3C_2T_x$(g) and SAED pattern(h). AFM image of monolayer $Ti_3C_2T_x$ nanosheets and the corresponding height measurement(i). XRD spectra of Ti_3AlC_2 and $Ti_3C_2T_x$ nanosheets(j). XPS spectra of $Ti_3C_2T_x$ nanosheets(k). High-resolution XPS spectra of Ti(l), C(m), O(n), F(o), Cl(p).

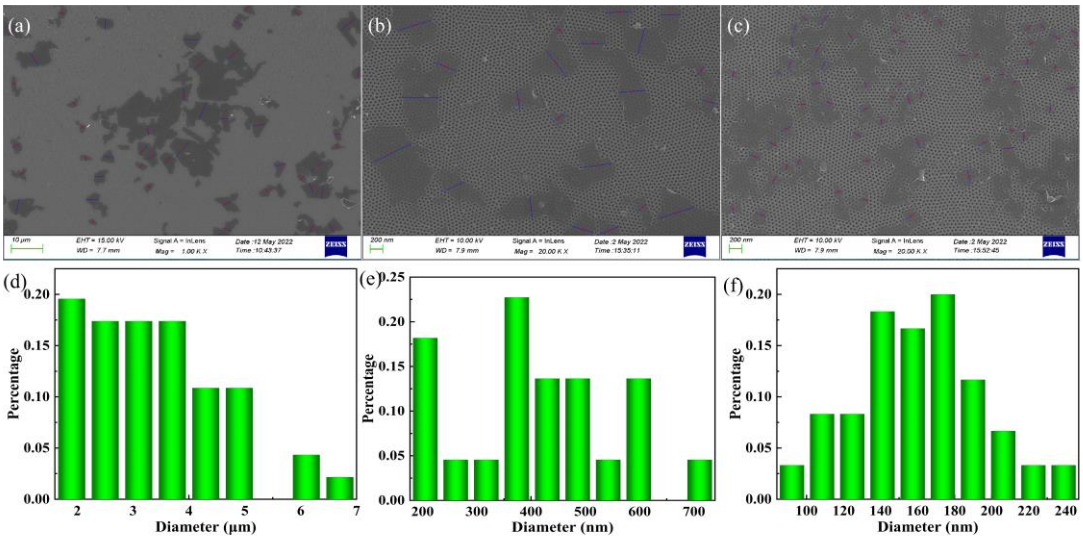

Figure S3 SEM images of $Ti_3C_2T_x$ ultrasound at 0 min(a), 15 min(b), and 30 min(c). The corresponding size statistics(d-f).

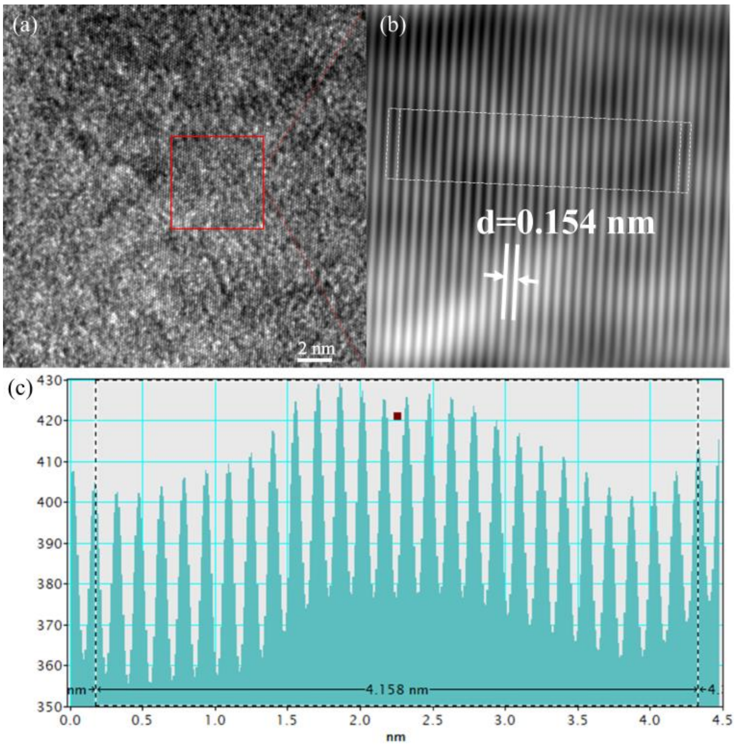

Figure S4 HRTEM images of $Ti_3C_2T_x$(a) and the corresponding Fourier transformation(b). Calculated interplanar crystal spacing for $Ti_3C_2T_x$(c).

2. Preparation of PVDF/$CsPbBr_3$ and PVDF/$CsPbBr_3$/$Ti_3C_2T_x$ composite nanofiber

The PVDF/$CsPbBr_3$/$Ti_3C_2T_x$ composite nanofiber were prepared by the electrospinning method. Here, N,N-dimethylformamide (DMF) was used as a solvent for the precursors. Initially, The $Ti_3C_2T_x$

suspension (solvent is DMF) was added to the DMF solution and sonicated for 30 min to obtain a uniformly dispersed solution. **Figure S5**a shows the optical images of different concentration $Ti_3C_2T_x$ in DMF solution at laser irradiation. It has Tyndal effect under laser in agreement with aqueous solution, proving that good dispersion of $Ti_3C_2T_x$ in DMF solution.[8] Further, We compared the UV-visible absorption spectra of $Ti_3C_2T_x$ in aqueous solution and DMF solution (**Figure S5**b). The similar absorption spectra indicated that $Ti_3C_2T_x$ is as well dispersed in DMF solution as in aqueous solution, which is also confirmed by the literature.[9] Subsequently, CsBr and $PbBr_2$ were added to the above solution and stirred for 20 min to dissolve it fully. The oleylamine (OAm) and oleic acid (OA) were added and stirred magnetically for 20 min at 500 rpm. Finally, PVDF (molecular weight 1200000) was added to obtain the precursor solution by magnetically stirring at 60 °C for 4 h with a weight concentration of 14%. Similarly, the PVDF/$CsPbBr_3$ precursors were prepared in the same way, except for the addition of $Ti_3C_2T_x$ in the first step. The optical images of precursors of PVDF/$CsPbBr_3$/$Ti_3C_2T_x$ and PVDF/$CsPbBr_3$ are shown in **Figure S6**.

The prepared precursor was drawn into a syringe with a 0.5 mm inner diameter stainless-steel needle for electrospinning. Electrospinning fibers were collected at a stainless-steel rotating cartridge collector with a speed of 3000 rpm. The distance from the needle to the collector is 18 cm. The flow rate was maintained at 0.04 mm/min. The applied voltage was set as 15 kV. Inside the electrospinning machine, the temperature is set to 30 °C and the humidity is adjusted at 60%. Then, the electrospinning film was dried for 2 h in a normal oven at 80 °C. Finally, the composite films with different $Ti_3C_2T_x$ contents were obtained.

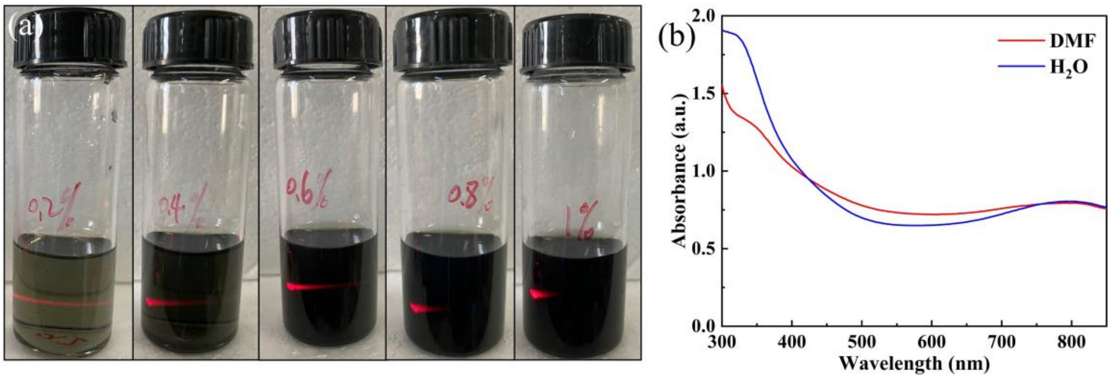

Figure S5 Optical images of $Ti_3C_2T_x$ dispersion in DMF solution under laser irradiation(a). UV−visible absorbance spectra of $Ti_3C_2T_x$ in DMF and H_2O (solutions diluted by a factor of 10)(b).

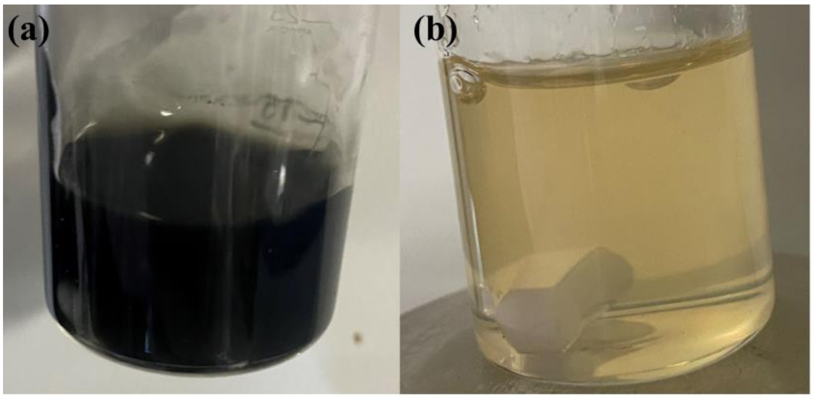

Figure S6 The optical images of precursors of PVDF/$CsPbBr_3$/$Ti_3C_2T_x$(a) and PVDF/$CsPbBr_3$(b).

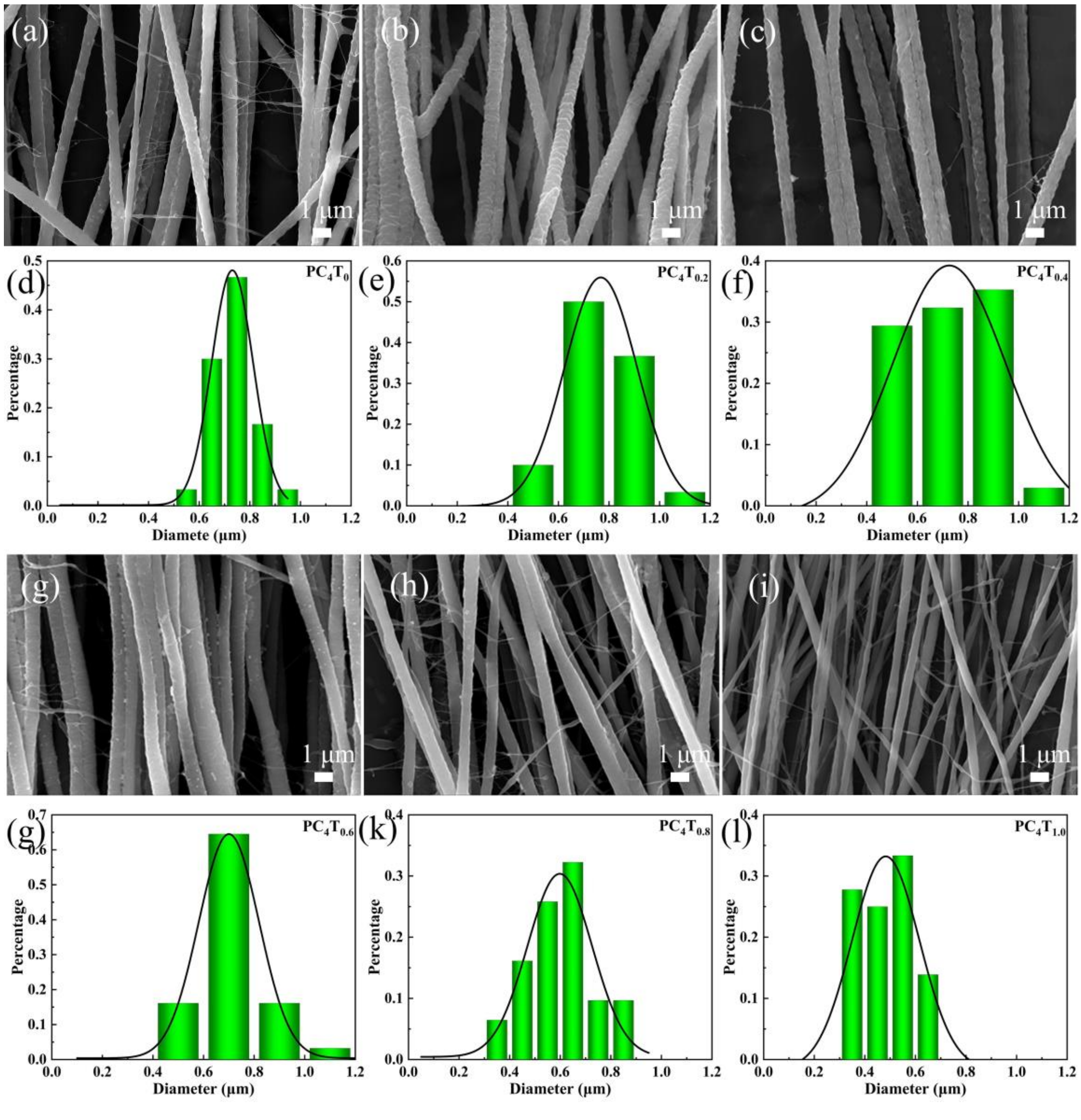

Figure S7 SEM images of composite fiber films(a-c,g-i) and corresponding size statistics (d-f, g-l).

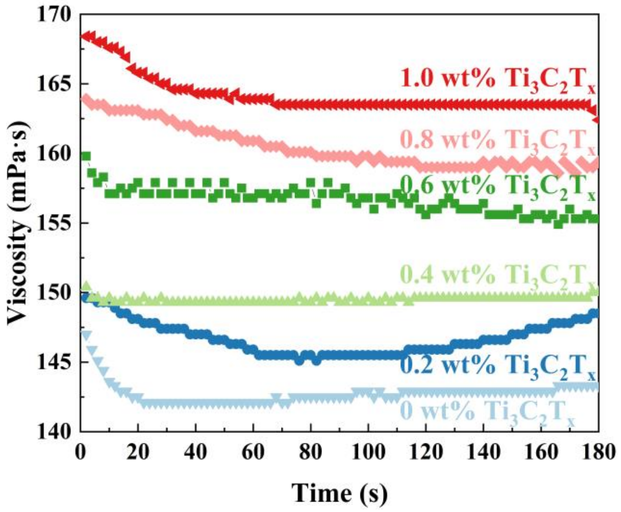

Figure S8 Viscosity tests of each component precursor.

The crystalline forms of PVDF and PVDF/4wt%$CsPbBr_3$ composite films are further explored by FTIR as shown in **Figure S9**. The α phase is represented by the vibrational bands at 762 cm^{-1} and 975 cm^{-1}, while the electroactive β phase is represented by the distinctive peaks at 840 cm^{-1}, 1275 cm^{-1}, and 1400 cm^{-1}.[10] The relative fraction of β phase can be calculated by Equation 1.[10b]

$$F_{(\beta)} = \frac{A_\beta}{\left(\frac{K_\beta}{K_\alpha}\right) A_\alpha + A_\beta}$$

Where A_β and A_α are the absorbances at 840 cm^{-1} and 762 cm^{-1}. K_β and K_α represent the absorption coefficients at the corresponding wavenumber, which are 7.7×10^4 and 6.1×10^4 cm^2mol^{-1}, respectively. The β phase contents of PVDF and PVDF/4wt%$CsPbBr_3$ composite film are 78.7% and 94.6%, respectively.

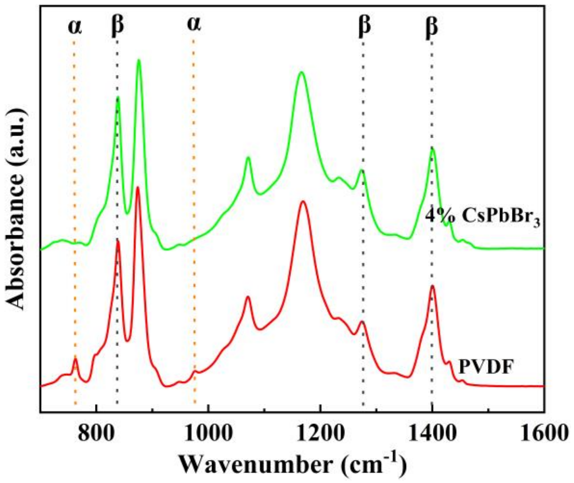

Figure S9 FTIR spectra of PVDF and PVDF/4wt%$CsPbBr_3$ composite film.

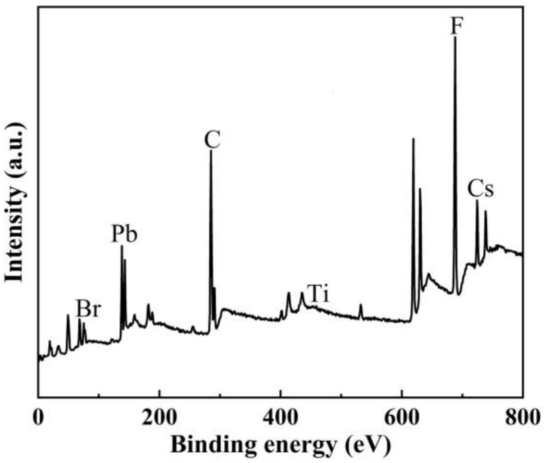

Figure S10 XPS spectra of PVDF/4wt%$CsPbBr_3$ composite film.

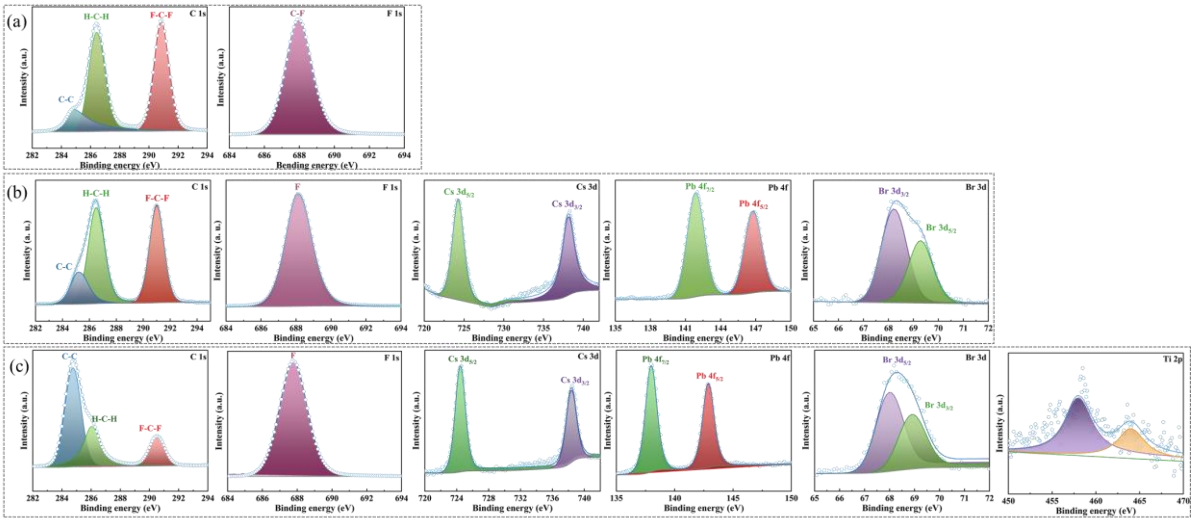

Figure S11 The high-resolution XPS spectra of PVDF(a), PVDF/$CsPbBr_3$(b), and PVDF/$CsPbBr_3$/$Ti_3C_2T_x$(c).

Since the voids and air gaps between the composite fibers will interfere with the measurement of d_{33}, the composite fibers are compressed into a cylinder using a mold, as reported in the literature[11]. The thickness of the cylinder is 0.2 mm (**Figure S12**). The samples are mounted on the meter and intermittently pressed by the meter's top head producing a direct reading, as shown in (**Figure S12**). The samples are tested at multiple points and the results are shown in **Table S1**. The average value is taken as the d_{33} value of samples. The d_{33} of PVDF/$CsPbBr_3$/$Ti_3C_2T_x$ composite film is 36.1 pC/N.

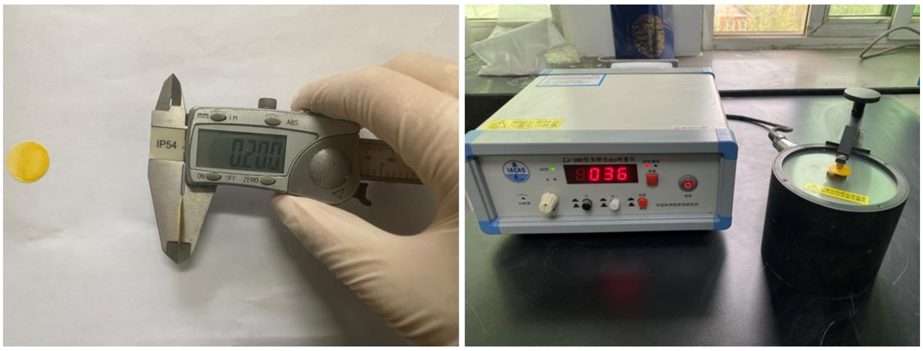

Figure S12 The thickness of the cylinder and d_{33} testing.

Table S1 Statistics of d_{33} measurements of samples.

Point	1	2	3	4	5	6	7	8	9	10
d_{33} (pC/N)	36	35	33	37	36	37	36	36	37	38

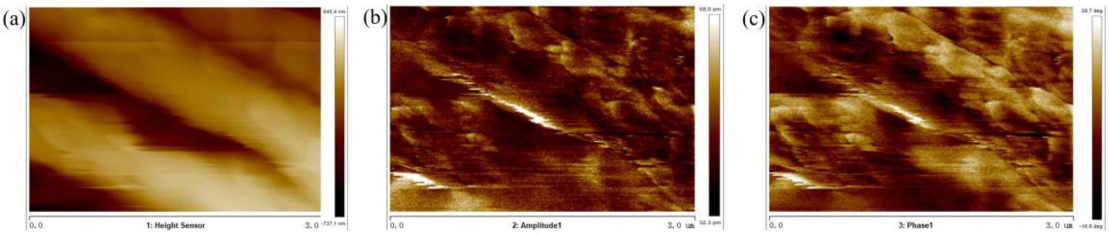

Figure S13 The topography(a), amplitude(b), and phase(c) images of PFM.

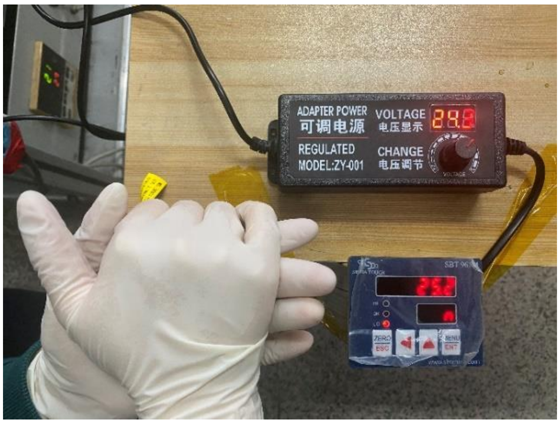

Figure S14 The value of the palm tap force testing.

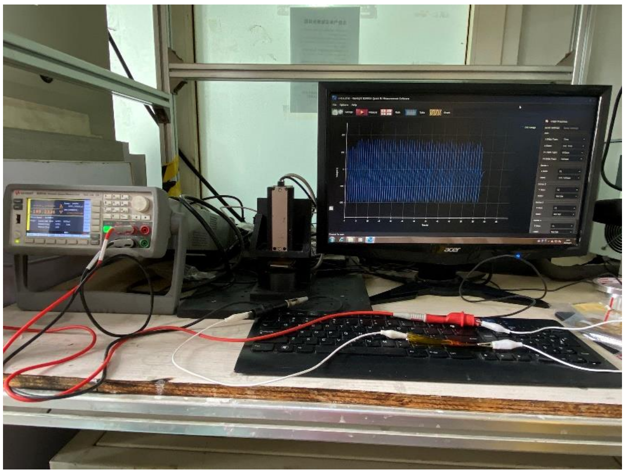

Figure S15 PENG performance testing system.

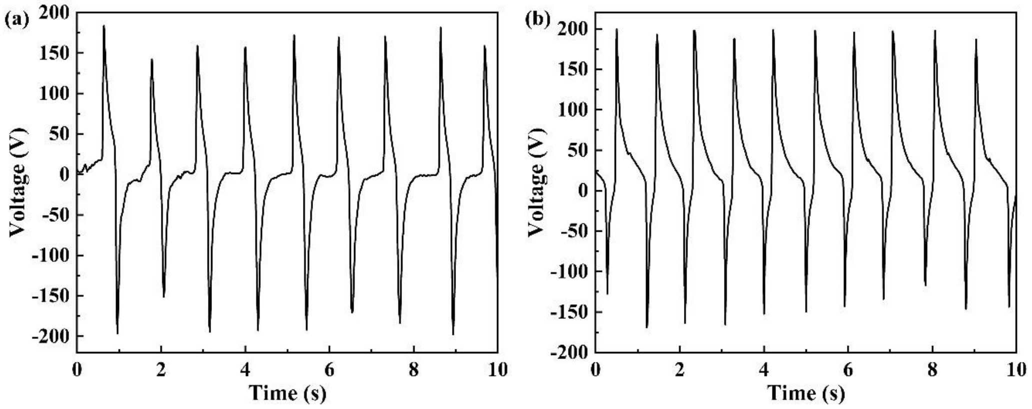

Figure S16 Piezoelectric output open-circuit voltage (V_{oc}) of PVDF/$CsPbBr_3$/$Ti_3C_2T_x$-PENG under forward(a) and reverse(b) connection.

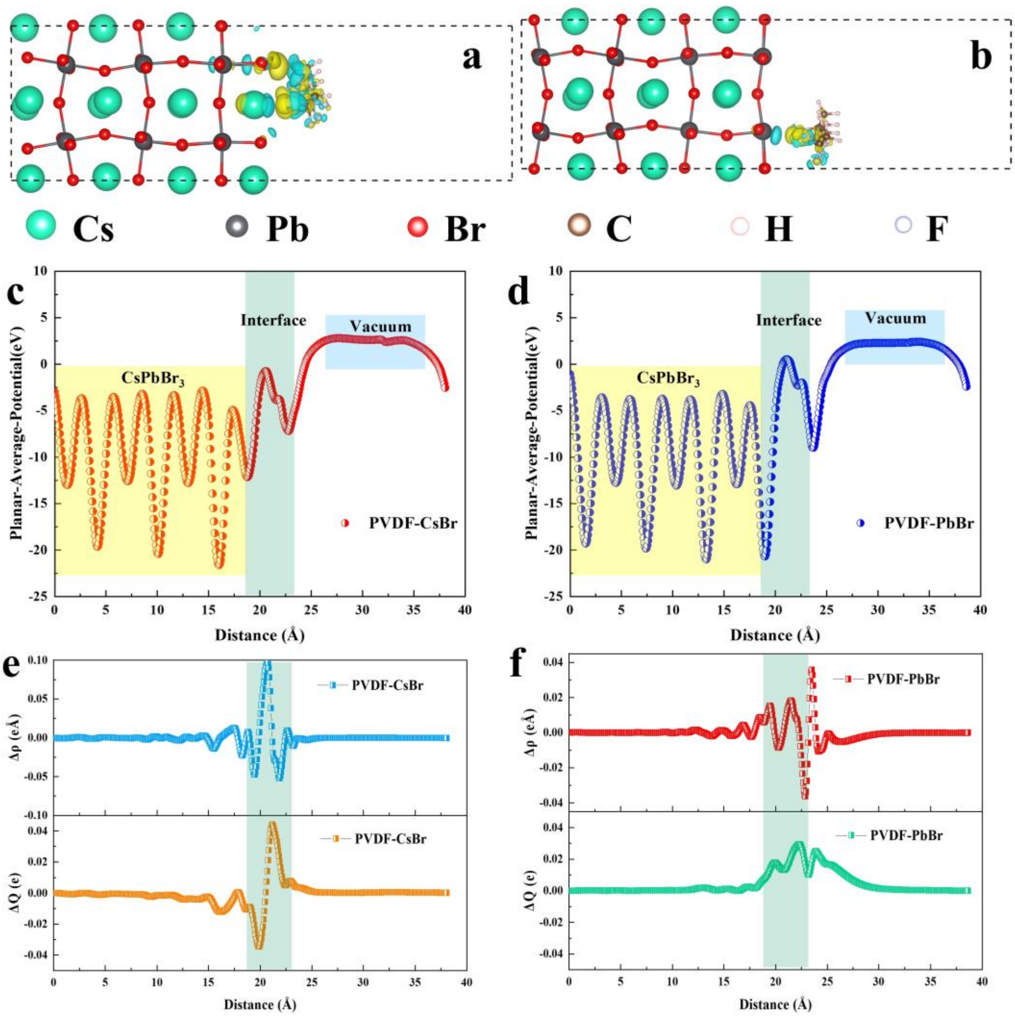

Figure S17 Interface induced charge density isosurface. PVDF-CsBr(a), PVDF-PbBr(b). Planar averaged electrostatic potential. PVDF-CsBr(c), PVDF-PbBr(d). Charge density difference along z axis and charge displacement curve of PVDF-CsBr(e), PVDF-PbBr(f).

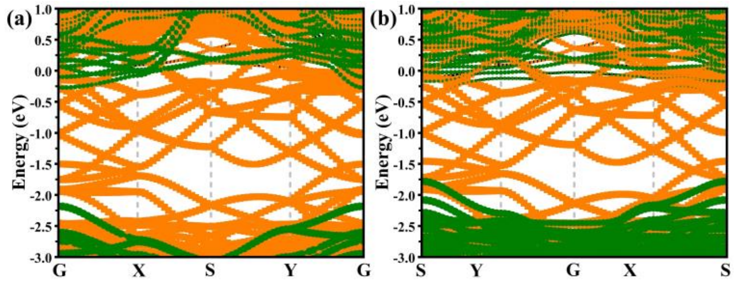

Figure S18 The heterostructure band structure of PVDF/$CsPbBr_3$/$Ti_3C_2T_x$. (a) Interface of CsBr-$Ti_3C_2T_x$. (b) Interface of PbBr-$Ti_3C_2T_x$. The $Ti_3C_2T_x$ band is plotted in orange curves, and the contribution of $CsPbBr_3$ is green.

References

[1] C. E. Shuck, K. Ventura-Martinez, A. Goad, S. Uzun, M. Shekhirev, Y. Gogotsi, *ACS Chem. Health Saf.* **2021**, 28, 326.

[2] M. Naguib, O. Mashtalir, J. Carle, V. Presser, J. Lu, L. Hultman, Y. Gogotsi, M. W. Barsoum, *ACS Nano* **2012**, 6, 1322.

[3] O. Mashtalir, M. Naguib, V. N. Mochalin, Y. Dall'Agnese, M. Heon, M. W. Barsoum, Y. Gogotsi, *Nat. Commun.* **2013**, 4, 1716.

[4] M. Naguib, M. Kurtoglu, V. Presser, J. Lu, J. Niu, M. Heon, L. Hultman, Y. Gogotsi, M. W. Barsoum, *Adv. Mater.* **2011**, 23, 4248.

[5] J. Halim, K. M. Cook, M. Naguib, P. Eklund, Y. Gogotsi, J. Rosen, M. W. Barsoum, *Appl. Surf. Sci.* **2016**, 362, 406.

[6] V. Natu, M. Benchakar, C. Canaff, A. Habrioux, S. Célérier, M. W. Barsoum, *Matter* **2021**, 4, 1224.

[7] a) M. Zhang, R. Liang, N. Yang, R. Gao, Y. Zheng, Y. P. Deng, Y. Hu, A. Yu, Z. Chen, *Adv. Energy Mater.* **2021**, 12; b) M. Li, J. Lu, K. Luo, Y. Li, K. Chang, K. Chen, J. Zhou, J. Rosen, L. Hultman, P. Eklund, P. O. A. Persson, S. Du, Z. Chai, Z. Huang, Q. Huang, *J. Am. Chem. Soc.* **2019**, 141, 4730.

[8] M. Naguib, Y. Gogotsi, *Acc. Chem. Res.* **2015**, 48, 128.

[9] K. Maleski, V. N. Mochalin, Y. Gogotsi, *Chem. Mater.* **2017**, 29, 1632.

[10] a) K. Ke, P. Pötschke, D. Jehnichen, D. Fischer, B. Voit, *Polymer* **2014**, 55, 611; b) P. Hu, L. Yan, C. Zhao, Y. Zhang, J. Niu, *Compos. Sci. Technol.* **2018**, 168, 327.

[11] M. Zhang, T. Gao, J. Wang, J. Liao, Y. Qiu, H. Xue, Z. Shi, Z. Xiong, L. Chen, *Nano Energy* **2015**, 11, 510.
